# Dielectric and Thermal Conductivity of Epoxy Resin Impregnated Nano-h-BN Modified Insulating Paper

**DOI:** 10.3390/polym11081359

**Published:** 2019-08-16

**Authors:** Hongda Yang, Qingguo Chen, Xinyu Wang, Minghe Chi, Heqian Liu, Xin Ning

**Affiliations:** 1 Heilongjiang Provincial Key Laboratory of Dielectric Engineering, School of Electrical and Electronic Engineering, Harbin University of Science and Technology, Harbin 150080, China; 2 Key Laboratory of Engineering Dielectrics and Its Application, Ministry of Education, Harbin University of Science and Technology, Harbin 150080, China; 3 State Grid Heilongjiang Electric Power Company Limited Electric Power Research Institute, Harbin 150030, China

**Keywords:** dry bushing, epoxy resin-impregnated paper, dielectric characteristics, thermal conductivity, space charge, nanocomposite

## Abstract

Epoxy resin-impregnated insulation paper (RIP) composites are used as the inner insulation of dry condenser bushing in the ultra-high voltage direct current (UHVDC) power transmission system. To improve the dielectric properties and heat conductivity of RIP, hexagonal boron nitride (h-BN) nano-flakes are added to the insulation paper at concentrations of 0–50 wt % before impregnation with pure epoxy resin. X-ray diffraction (XRD), scanning electron microscopy (SEM) observations, thermal conductivity as well as the typical dielectric properties of direct current (DC) volume conductivity. DC breakdown strength and space charge characteristics were obtained. The maximum of nano-h-BN modified heat conductivity reach 0.478 W/(m·K), increased by 139% compared with unmodified RIP. The DC breakdown electric field strength of the nano-h-BN modified RIP does not reduce much. The conductivity of nano-h-BN modified is less sensitive to temperature. As well, the space charge is suppressed when the content is 50 wt %. Therefore, the nano-h-BN modified RIP is potentially useful in practical dry DC bushing application.

## 1. Introduction

As people′s demand for electricity grows, transmission systems are moving toward high-capacity and high-voltage. In recent years, HVDC transmission has been favored by the power industry. It can easily adjust the direction of the power flow, does not produce low-frequency oscillation, has no stability constraints, and does not increase the short-circuit capacity [1]. Especially in the long distance, and large capacity transmission, high voltage DC transmission has obvious advantages. As one of the key equipment of HVDC transmission system, the safe and stable operation of HVDC bushing is very important to HVDC transmission system. HVDC bushing can be divided into oil-paper insulated bushing and epoxy resin-paper dry DC bushing according to the type of insulation. Compared to oil-paper insulated bushing, dry DC bushing has excellent mechanical properties and heat resistance, while avoiding secondary hazards such as explosion and fire caused by faults. In addition, it also has the advantages of light weight, flexible installation angle and environmental protection insulating material [2]. The capacitor core of a high-voltage dry DC bushing is made by alternately rolling a multilayered crepe paper and aluminum foil around the center conductor, vacuum-impregnating the paper with epoxy resin and applying a staged curing process [3]. Epoxy resin-impregnated paper (RIP) is the main insulation. The dielectric property of RIP is crucial for safe and stable operation of DC dry bushing. While space charge will inject into epoxy RIP composite under DC electric field. The space charge in RIP will make the electric field distribution in the bushing distort and local electric field become larger [4]. The aging of insulation under high electric field intensity for a long time will accelerate, which seriously affects the safe and stable operation of the bushing. Wu Kai et al. studied the space charge characteristics of RIP [2]. But no method for suppressing space charge is proposed. On the other hand, large current will make the center conductor generate heat. As well, the thermal conductivity of RIP is poor, the heat of the inner layer insulation cannot be effectively dissipated [5]. When the bushing operates for a long time, there will be a large temperature gradient between the inner insulation and the outer insulation. Numerous studies have shown that DC conductivity is affected by temperature. Electric field distribution is affected by DC conductivity under DC electric field. In summary, the electric field distribution of the high voltage DC bushing can be improved from the following aspects. One is to suppress space charge injection. Reduce the influence of space charge on the internal electric field distribution of RIP; the other is to reduce the dependence of RIP conductivity on temperature and improve thermal conductivity of RIP. Firstly, the reduced RIP conductivity dependence on temperature can reduce the difference in conductivity between the inner insulation and the outer insulation when the bushing works under the temperature gradient. Secondly, the increase of RIP thermal conductivity can reduce the temperature gradient between inner insulation and outer insulation. A lot of research on space charge suppression by nano-fillers has been carried out [6,7,8,9,10]. And a large number of studies have shown that doping high thermal conductivity fillers can improve thermal conductivity [11,12,13,14]. Most of the research on thermal conductivity focuses on nano-modification of epoxy resins and other polymers. However, nano-fillers can increase the viscosity of epoxy resin. The epoxy resin with high viscosity is harmful to the impregnation of insulating paper, which affects the insulation performance of RIP. Therefore, nanofiller-modified epoxy resin is not suitable for RIP composites. There are also some studies on nano-micron filled modified oil-impregnated paper and some other functional papers [15,16,17,18,19,20]. However, few studies have investigated nano-modified RIP composites. Only Chen et al. studied the dielectric properties of nano-SiO_2_ modified RIP [1].

Because hexagonal boron nitride (h-BN) has high thermal conductivity and excellent dielectric properties. In this paper, nano-h-BN modified insulating paper impregnated with unmodified epoxy resin was used to test its thermal conductivity, DC conductivity, DC breakdown field strength and space charge characteristics. The results show that the nano-h-BN modified RIP has excellent thermal conductivity, space charge suppression and less temperature dependence than unmodified RIP.

## 2. Materials and Methods

### 2.1. Sample Preparation

**Figure 1:**
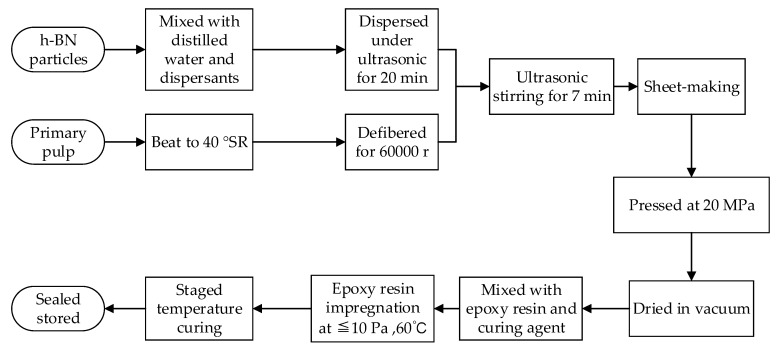
The flow chart of the making process of epoxy resin impregnated nano-h-BN modified pressboard.

The nano-h-BN modified pressboards were composed of unbleached coniferous kraft pulp, distilled water (µ < 10 S/cm), h-BN nano-flakes (150624) with lamellar structure, with average diameter of 0.5 μm, thickness <100 nm, purity >99%, purchased from Peng Da Technology Co., Ltd. (Yingkou, China). Epoxy resin and curing agent for impregnation are the WSR618 (E-51) matrix purchased from Xingchen Synthetic Material Co., Ltd. (Nantong, China) and methyl hexahydrophthalic anhydride (MHHPA) was bought from Huicheng Electronic Materials Co., Ltd. (Puyang, China). 2,4,6-Tri(dimethylaminomethyl)phenol (DMP-30, Shanfeng Chemical Co., Ltd., Changzhou, China) was used as an accelerant. According to the industrial manufacturing process of insulation pressboards, the samples were made through the seven steps of pulping, doping (with 0, 2.5, 5, 10, 20, 40 or 50 wt % nano-h-BN), shaping, compressing, drying, vacuum impregnating with epoxy resin and staged temperature curing. These processes used a beater (TD 6-23, Tongda Light Power Equipment Co., Ltd., Xianyang, China), ultrasonic dispersion instrument (JP-020, Jiemeng Cleaning Equipment Co., Ltd., Shenzhen, China), standard agitator (DJ1C-100, Dadi Automation Instrument, Jintan, China), handsheet former (TD10-200, Tongda Light Power Equipment Co., Ltd., Xianyang, China), curing press (XLB25-D, Shuangli Automation Technology Equipment Co., Ltd., Huzhou, China), and vacuum drying chamber (DZF-6210D, Haixiang Instrument and Equipment Factory, Shanghai China), as shown in Figure 1, in which SR is the unit of beating degree [16].

Polyethylene glycol (PEG, Tianjin Guangfu Chemical Research Institute, Tianjin, China) was used as a modifier to avoid aggregation of nano-h-BN. The long-chain structure of PEG has a location-obstructing effect that can prevent the aggregation of nano-h-BN in suspension [16]. Moreover, the fine combination with cellulose and retention of nano-h-BN flakes was guaranteed by the twining effect from the long-chain structure of PEG [21]. Finally, the epoxy resin impregnated nano-h-BN modified pressboard is obtained. 

**Figure 2:**
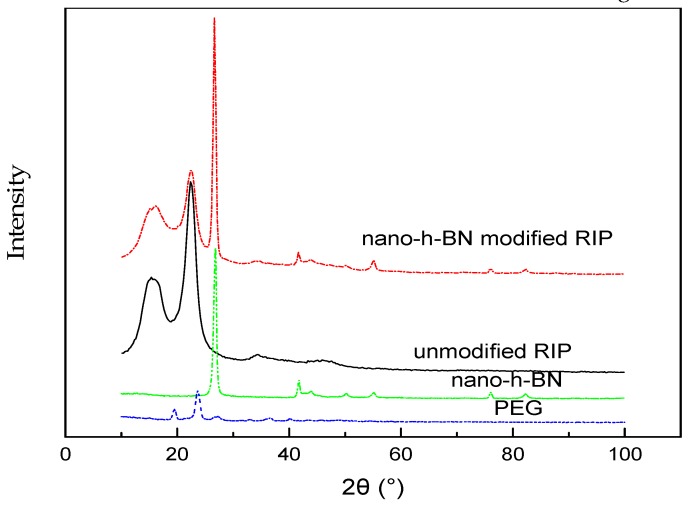
XRD (X-ray diffraction) spectra of nano-h-BN, PEG, unmodified and nano-h-BN modified RIP (epoxy resin-impregnated insulation paper).

The X-ray diffraction (XRD, Empyrean Ruiying, PANalytical B.V., Netherlands) curves of nano flakes, PEG, non-modified RIP and nano-h-BN modified RIP are shown in Figure 2.

Figure 2 shows that the characteristic peaks in curve of the nano-h-BN modified RIP is identical to these of both unmodified RIP and nano-h-BN. Besides, there is no other characteristic peak, which suggests that the addition of PEG does not introduce by-products.

**Figure 3:**
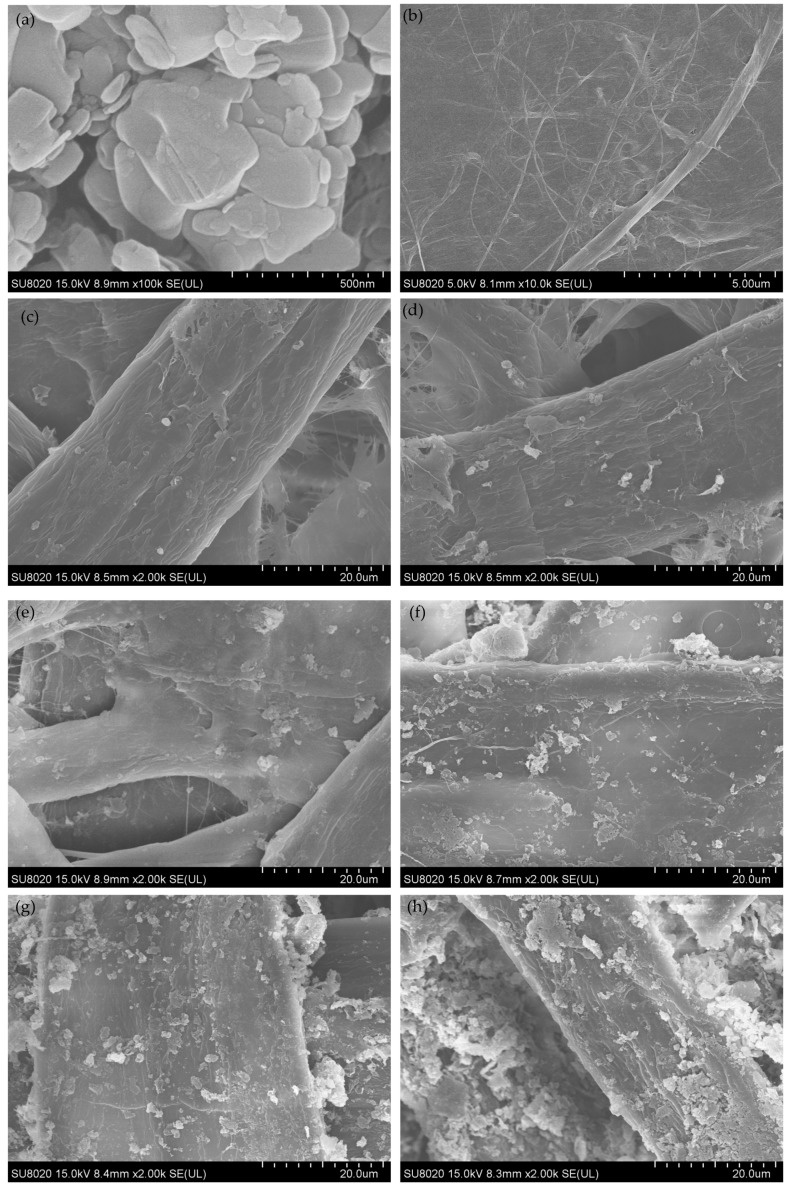
(**a**) SEM micrographs of nano-h-BN (**b**) SEM micrographs of unmodified pressboard; (**c**) SEM micrographs of modified pressboard with 2.5 wt % h-BN; (**d**) SEM micrographs of modified pressboard with 5 wt % h-BN; (**e**) SEM micrographs of modified pressboard with 10 wt % h-BN; (**f**) SEM micrographs of modified pressboard with 20 wt % h-BN; (**g**) SEM micrographs of modified pressboard wit 40 wth % h-BN; (**h**) SEM micrographs of modified pressboard with 50 wt % h-BN;.

The microstructures of nano-h-BN flakes, nano-h-BN modified and unmodified pressboards are observed using scanning electron microscopy (SEM, SU8020 Hitachi High Technologies Corp, Tokyo, Japan) shown in SEM micrographs of Figure 3.

### 2.2. Measurement System

The thermal conductivity characteristics of non-modified and nano-h-BN-modified RIP were tested by thermal conductivity tester (DTC-300, TA Instruments, Newcastle, PA, USA), the standard ASTM-E1530 was applied during the thermal conductivity tests. The test temperature is 298.15 K. The diameter of the samples are 50 mm and the thickness is about 1 mm [22].

The DC conductivity characteristics of non-modified and nano-h-BN-modified RIP were studied at the temperature of 298.15, 323.15 and 348.15 K by measuring the leakage current with a three-terminal electrode system placed in an oven. The system was connected to an electrometer (6517A, Keithley Instruments, Inc., Cleveland, OH, USA). And an electrical field of 1–15 kV/mm was applied to the sample by DC high-voltage generators. Aluminum was evaporated onto the surface of the sample as electrodes. Setting the test temperature and waiting 60 min make the temperature of the samples stable. The stable current (*I*) was recorded after applying the DC voltage for 10 min. Four samples of the same concentration produced in the same batch were randomly sampled for conductivity testing. The average conductivity of the four samples represents the conductivity of the sample with this concentration. 

In addition, high-voltage generators and column polar structure, in compliance with the standard ASTM-D149 were applied during the DC breakdown strength tests, and the entire testing system was placed in transformer oil, at the temperature of 298.15 K [23]. Both the height and the diameter of the electrode is 25 mm. The thickness at the breakdown point was measured for calculation. To reduce the influence of data scattering, the average value of multiple measuring data was taken.

**Figure 4:**
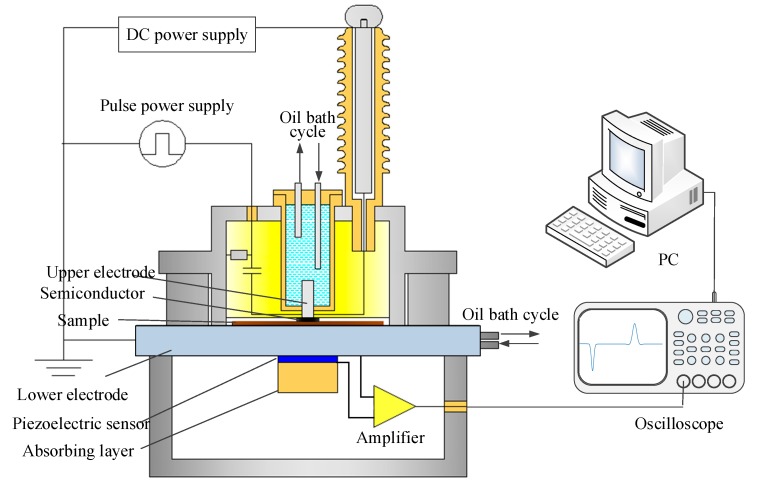
Schematic of space charge measurement system.

The space charge measurements were achieved with the pulsed electro-acoustic (PEA) technique. The electrodes of PEA test bench are made of aluminum for the electrode next to the acoustic sensor and a polymer filled with carbon black for the other electrode. The acoustic couplings at both interfaces of the sample were obtained with silicone oil. The effective surface area of probing, S, is about 2 cm^2^. The PEA pulse source giving a pulse field of 2 kV/mm. The electrical field of test is 10 and 20 kV/mm respectively. The test temperature is 298.15 K. The setup of the space charge measurement system is shown in Figure 4.

## 3. Results

### 3.1. Thermal Conductivity Characteristics of Non-Modified and Nanno-h-BN-Modified RIP

**Figure 5:**
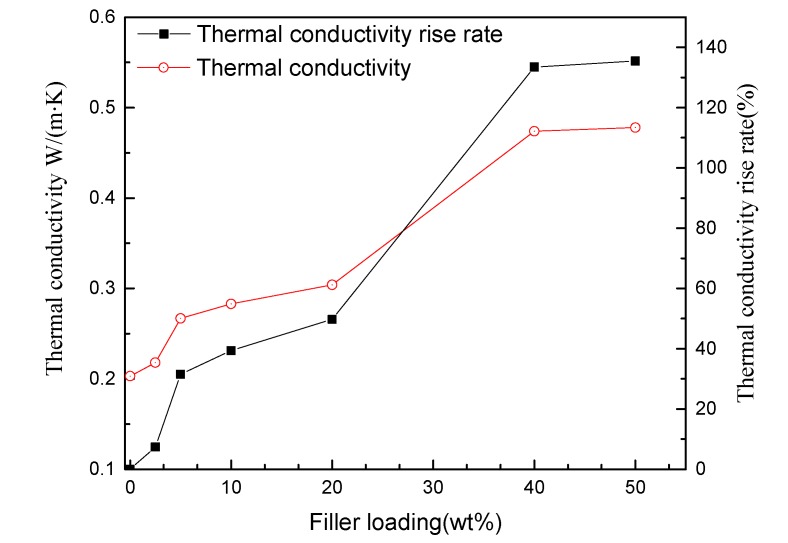
Thermal conductivity versus nano-h-BN content of RIP.

The relationship between thermal conductivity and the nano-h-BN content of RIP is shown in Figure 5. It shows that the thermal conductivity of nano-h-BN modified RIP increases with the increase of nano-h-BN content. The thermal conductivity of unmodified RIP is 0.203 W/(m·K) and when the nano-h-BN content is 50 wt % the thermal conductivity reaches the maximum value 0.478 W/(m K). Compared with unmodified RIP the maximum thermal conductivity rise rate is 139%. In detail, when the filler loading less than 20 wt % the thermal conductivity raises slowly; when the filler loading between 20 and 40 wt % the thermal conductivity grow rapidly; while the filler loading reaches 40 wt % the thermal conductivity hardly grow with filler content.

Thermal conductivity rise rate defined as *η*:

(1) $$\eta =(\lambda_{m}-\lambda_{0})/\lambda_{0}\times 100\%$$

Among them, *λ_m_* is the thermal conductivity of nano-h-BN modified RIP when the filler loading is m wt %, *λ*_0_ is the thermal conductivity of unmodified RIP.

### 3.2. DC Conductivity Characteristics of Unmodified and Nano-h-BN-Modified RIP

**Figure 6:**
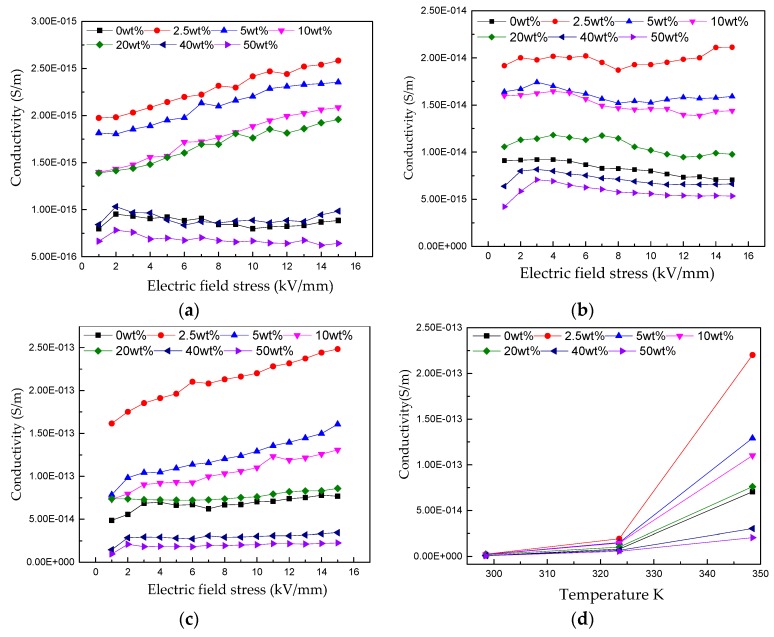
(**a**–**c**) are respectively the DC conductivity versus E curves of RIP with different h-BN nano-flakes components at 298.15, 323.15 and 348.15 K; (**d**) is DC conductivity versus temperature of RIP with different h-BN nano-flakes components at 10 kV/mm.

The relationship between conductivity (γ) and electric field stress (*E*) of RIP with different nano-h-BN contents are shown in Figure 6. Figure 6a–c show that the conductivities of nano-h-BN_-_modifies RIP at 298.15, 323.15, 348.15 K are all first increase and then decreased with the nano-h-BN content increasing. As well, the conductivity of RIP increases slightly with increasing of electrical field stress; Figure 6d shows the conductivity increases with temperature rising, at 10 kV/mm. In detail, the conductivity of 40 and 50 wt % RIP increases less with temperature rising than that of unmodified RIP.

To observe the change of DC conductance with temperature intuitively, define the DC conductivity rise multiple as α:

(2) $$\alpha =(\gamma_{t_{2}}-\gamma_{t_{1}})/\gamma_{t_{1}}$$

Among them, *γ*_*t*1_ and *γ*_*t*2_ are the DC conductivity at temperatures *t*_1_ and *t*_2_, respectively. The value of α under different temperature changes shows in Table 1:

Table 1 shows temperature changes from 298.15 to 323.15 K, α of nano-h-BN modified RIP are all smaller than unmodified RIP; In addition, temperature changes from 323.15 to 348.15 K and 298.15 to 348.15 K, all the α of nano-h-BN modified RIP are all smaller than unmodified RIP, except 2.5 wt % modified RIP. As well, α decrease with the increase of nano-h-BN content. It means that as the h-BN content increases the conductivity is less sensitive to temperature.

### 3.3. DC Breakdown Strength Characteristics of Naon-Modified RIP

**Figure 7:**
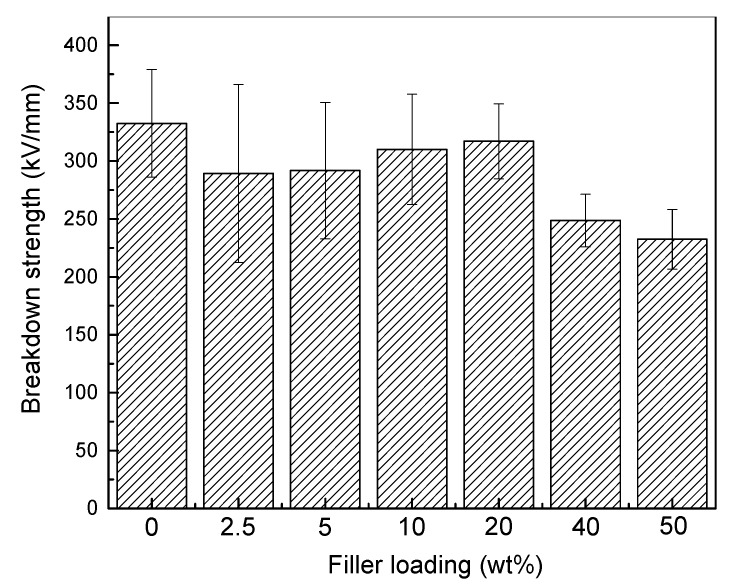
Breakdown strength histogram (mean and standard deviations) of RIP with different h-BN nano-flakes components.

The DC breakdown strength characteristics of modified and unmodified RIP are shown in Figure 7. When the filler content less than 20 wt % the DC breakdown stress of nano-h-BN-modified RIP does not decrease much compared with unmodified RIP. While when the filler loading reaches 40 and 50 wt % the DC breakdown stress are 25.25% and 30.13% down respectively. On the whole, h-BN has little effect on breakdown, and does not significantly deteriorate the insulation. 

### 3.4. Space Charge Characteristics of Unmodified and Nano-h-BN-Modified RIP

**Figure 8:**
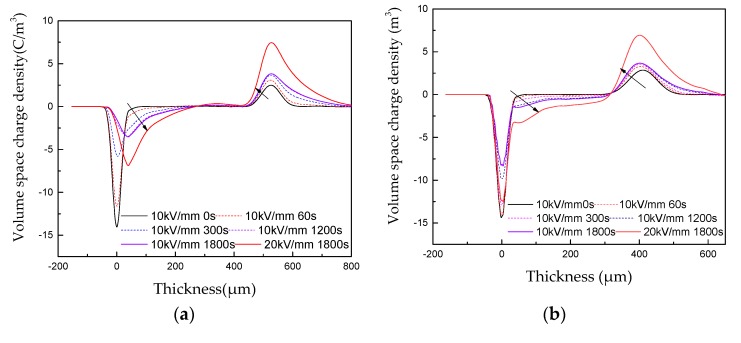

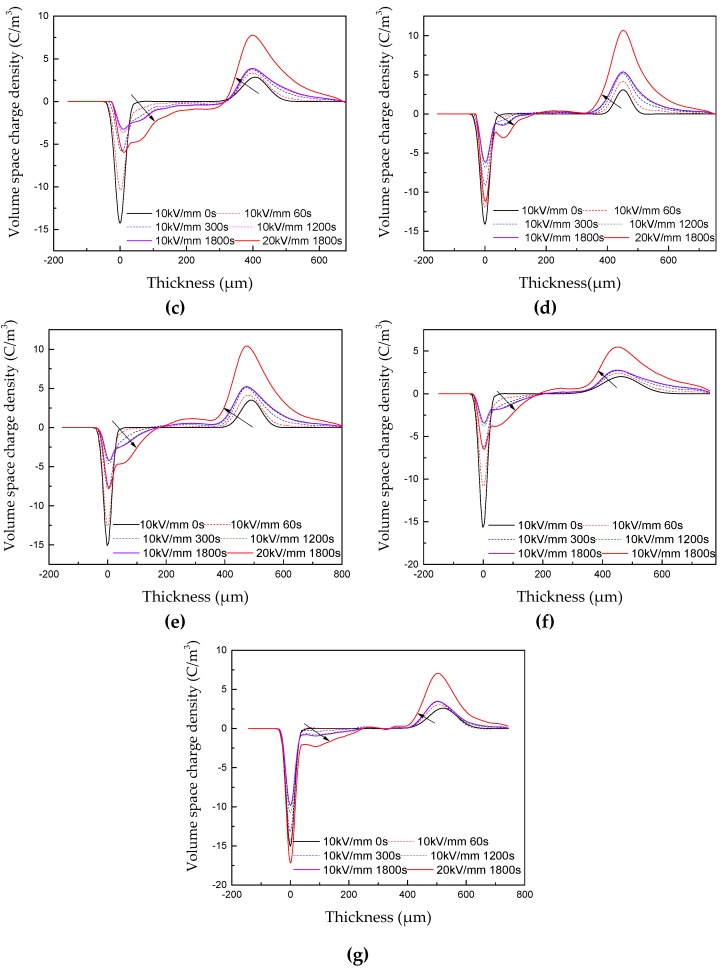
(**a**–**g**) are respectively the Space charge Characteristics of modified RIP with 0 wt %, 2.5 wt %, 5 wt %, 10 wt %, 20 wt %, 40 wt % and 50 wt % h-BN.

The space charge Characteristics of unmodified and nano-h-BN-modified RIP is shown in Figure 8. Figure 8 shows the space charge in RIP samples increases with time at the same electrical field. And, the space charge tends to be stable at 1800 s. In addition, when the applied voltage time is 1800 s and the electric field is 20 KV/mm, the space charge density is greater than 10 kV/mm. Moreover, for RIP samples with different concentrations, the injected charge is homopolarity. The injected charge moves to the other electrode in the form of a charge packet; Figure 8a shows that charge peak at cathode interface moves inside the sample compared with Figure 8b–f. Figure 8a–c show the space charge injection in 2.5 and 5 wt % nano-h-BN modified RIP is deeper than unmodified RIP. Figure 8a,d,e,f show 10, 20 and 40 wt % nano-h-BN modified RIP injected less space charge than unmodified RIP at the cathode, while more space charge injected at the anode. Figure 8a,g show 50 wt % RIP sample injected less space charge at the cathode than unmodified RIP, and no increase in anode. In another word, 50 wt % nano-h-BN modified RIP can suppress space charge injection.

## 4. Discussion

**Figure 9:**
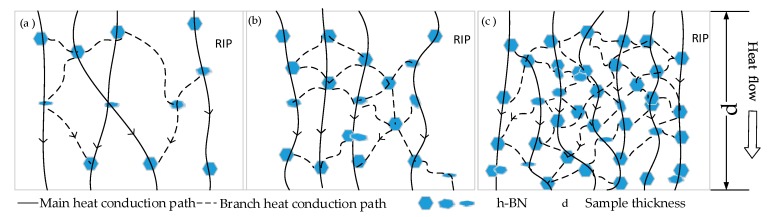
(**a**) h-BN modified pressboard with low filler loading (wt %); (**b**) h-BN modified pressboard with medium filler loading (wt %); (**c**) h-BN modified pressboard with high filler loading (wt %).

For heat conduction, heat conduction schematic diagram of nano-h-BN modified RIP with different nano-flakes components are shown in Figure 9. Nano-h-BN flakes with high heat conductivity [12]. When the nano-h-BN loading is low (0–2.5 wt %), the heat conductivity increases slightly. Owing to low h-BN nano-flakes loading (wt %) in RIP, the heat conduction network is incomplete shows as Figure 9a. A few heat conduction path formed in nano-modified-RIP. As a result, the heat conductivity is not increase much; when the h-BN nano-flakes between 2.5–20 wt %, there are more h-BN nano-flakes in RIP. More heat conduction path formed in RIP, as Figure 9b shows. So, the heat conductivity increases a lot and the η reached about 50% when the nano-h-BN is 20 wt %. When the nano-h-BN loading between 20–50 wt % both main and branch heat conduction path significant increase. Therefore, the heat conductivity significant increase at the nano-h-BN loading is 20–50 wt %. While the nano-h-BN loading from 40 to 50 wt % the heat conductivity hardly changed. Since the heat conduction paths are almost saturated. The heat conductivity increases slowly or stops growth as the filler loading continuous increase, when the nano-h-BN over 40 wt %. 

For the DC conductivity nano-h-BN in RIP affects charge transport in two ways. One is the nano-h-BN can reduce the distance between cellulose, can make the transport of carriers become easier. Another, nano-h-BN can scatter carriers, makes carrier transport more difficult [24]. As well, scattering effect of nano-h-BN increases with the increase of its content. When the nano-h-BN content between 0–20 wt %, the affection of reduce distance between cellulose is dominant. While the scattering become stronger with the increasing of nano-h-BN content. As a result, the DC conductivity of nano-h-BN modified RIP is higher than unmodified RIP, when the content of nano-h-BN between 0–40 wt %. And the DC conductivity of RIP decreases with the increase of nano-h-BN content; The scattering effect is dominant when the nano-h-BN content reach 50 wt %, the DC conductivity of 50 wt % modified RIP is lower than the DC conductivity of unmodified RIP. Besides, the temperature rise can make carrier get more energy. With the increase of temperature, the emittance of thermally assisted electrons in the electrode field increases, and more electrons are injected into the dielectric, which results in an increase in the number of carriers that can participate in the conductance, and the conductivity of RIP increases obviously [6]. The transport of carrier becomes easier. As a result, the DC conductivity increase with the temperature rise.

Sensitivity of DC conductivity to temperature. Ionic conductivity dominates the conductivity of composite dielectrics in low field strength region [6]. As well, its conductivity γ changes with temperature is in accordance with the following law:

(3a) $$\gamma =Ae^{-B/T}$$

Take the logarithm of Equation (3a):

(3b) lnγ=lnA−B/T

Among them, A and B are constant.

**Figure 10:**
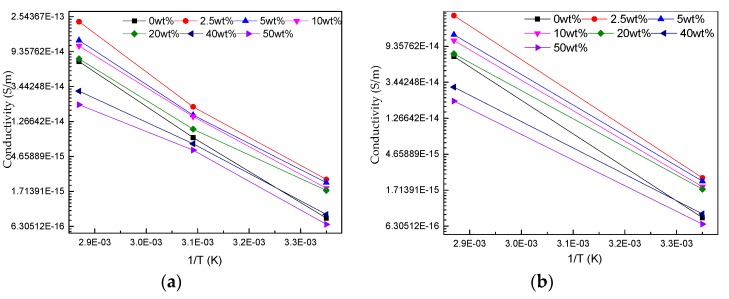
(**a**) The relationship of lnγ and 1/T (temperature change from 298.5 to 323.5 K and 323.5 to 348.5 K); (**b**) The relationship of lnγ and 1/T (temperature change from 298.5 to 348.5 K).

According to Equation (3b), B is obtained under diffident temperature changes, as shown in Table 2. And the relation of ln γ and 1/T show in Figure 10.

The trend of B with temperature is similar to that of α. And the B is the slope of Figure 10.

Combined with the Table 2 and Figure 10, the conductivity of nano-h-BN modified RIP is less sensitive to temperature. The reason for it maybe the temperature rise makes the carrier transport accelerate, which makes the scattering of nano-h-BN becomes stronger, the carrier transport becomes harder. 

For the change of DC breakdown strength with nano-h-BN content, it is closely to its DC conductivity. The DC conductivity can reflect the transport of carrier at a macroscopic level. Figure 6a shows that the DC conductivity increase first and then decrease with the increase of nano-h-BN content (0–20 wt %). As well, the DC breakdown the same tend to the DC conductivity, when the nano-h-BN content is 0–20 wt %. Owing to the increase of DC conductivity makes the carrier transport easier, the free path become longer. As a result, the DC breakdown decrease. However, with the increase of the content of nano-h-BN, its scattering becomes stronger and the DC conductivity decrease, the DC breakdown increase slightly. The scattering further strengthening, with the increase of the nano-h-BN (20–50 wt %), the DC conductivity continuous decline. Also, the DC conductivity of 50 wt % RIP is lower than unmodified RIP. While with the increase of increase of nano-h-BN content, nano-BN flakes are agglomerated. As well, some defects, such as micropore, are introduced into RIP. Therefore, the DC breakdown is reduced [25]. 

Space Charge Characteristics of RIP, due to the higher DC conductivity of 2.5 and 5 wt % RIP and the weak scattering of nano-h-BN, carrier transport easier. As a result the charge injection depth is greater than unmodified RIP. The charge injected into the cathode continuously moves towards the anode and neutralizes near the anode. With the increase contents (10–40 wt %) of nano-h-BN the scattering become stronger. The charge injection at cathode is decline, but it increases at anode. It may be the injected charge of the cathode is blocked and cannot be neutralized near the anode. When the content is 50 wt %, due to the strong scattering the DC conductivity is lower than unmodified RIP. The strong scattering enhances charge injection barrier. Space charge injection is suppressed when the nano-h-BN content is 50 wt %.

## 5. Conclusions

Based on the experimental study on the dielectric properties of nano-h-BN modified RIP, the following conclusions have been drawn:

1. Nano-boron nitride can improve the thermal conductivity of RIP while maintaining low DC conductivity and high DC breakdown strength performance;
2. Proper concentration of nano-h-BN can reduce the sensitivity of RIP conductance to temperature;
3. 50 wt % nano-h-BN modified RIP inhibits space charge compared with unmodified RIP.

## Figures and Tables

**Table 1:** α under different temperature changes.

Temperature (K)	0 wt %	2.5 wt %	5 wt %	10 wt %	20 wt %	40 wt %	50 wt %
298.15→323.15	9.03	7.77	5.92	6.74	4.78	6.56	7.37
323.15→348.15	7.80	10.41	7.48	6.54	6.46	3.50	2.66
298.15→348.15	87.24	90.11	57.63	57.37	42.16	33.02	29.65

**Table 2:** B under different temperature changes.

Temperature (K)	0 wt %	2.5 wt %	5 wt %	10 wt %	20 wt %	40 wt %	50 wt %
298.15→323.15	8904	8023	7469	7905	6779	7814	8205
323.15→348.15	9808	10980	9639	9110	9064	6783	5855
298.15→348.15	9321	9387	8471	8461	7833	7338	7121
